# Using supervised machine-learning approaches to understand abiotic stress tolerance and design resilient crops

**DOI:** 10.1098/rstb.2024.0252

**Published:** 2025-05-29

**Authors:** Rajneesh Singhal, Paulo Izquierdo, Thilanka Ranaweera, Kenia Segura Abá, Brianna N.I. Brown, Melissa D. Lehti-Shiu, Shin-Han Shiu

**Affiliations:** ^1^ Department of Plant Biology, Michigan State University, East Lansing, MI 48824, USA; ^2^ DOE Great Lakes Bioenergy Research Center, Michigan State University, East Lansing, MI 48824, USA; ^3^ Genetics and Genome Sciences Program, Michigan State University, East Lansing, MI 48824, USA; ^4^ Department of Computational Mathematics, Science, and Engineering, Michigan State University, East Lansing, MI 48824, USA

**Keywords:** climate change, machine learning, resilient crops, abiotic stress

## Abstract

Abiotic stresses such as drought, heat, cold, salinity and flooding significantly impact plant growth, development and productivity. As the planet has warmed, these abiotic stresses have increased in frequency and intensity, affecting the global food supply and making it imperative to develop stress-resilient crops. In the past 20 years, the development of omics technologies has contributed to the growth of datasets for plants grown under a wide range of abiotic environments. Integration of these rapidly growing data using machine-learning (ML) approaches can complement existing breeding efforts by providing insights into the mechanisms underlying plant responses to stressful conditions, which can be used to guide the design of resilient crops. In this review, we introduce ML approaches and provide examples of how researchers use these approaches to predict molecular activities, gene functions and genotype responses under stressful conditions. Finally, we consider the potential and challenges of using such approaches to enable the design of crops that are better suited to a changing environment.

This article is part of the theme issue ‘Crops under stress: can we mitigate the impacts of climate change on agriculture and launch the ‘Resilience Revolution’?’.

## 1. Introduction

By 2050, the human population is projected to reach 10 billion, requiring a 35−56% increase in food production compared with 2010 [1,2]. Meeting this demand through traditional means would necessitate cultivating an additional area twice the size of India [1]. This challenge is further exacerbated by the threats to food production posed by climate change: average global temperatures have risen by 1.7°C and CO_2_ levels by 50% (from 280 to 420 parts per million) since the mid-1700s [3,4], with projections of a further 3−5°C increase in temperature by 2100 and an increase in CO_2_ to 550 parts per million by 2050 if no action is taken [5,6]. This warming has resulted in more frequent and severe abiotic stresses, such as drought, high temperatures and flooding. While individual stresses can have varying effects on crop yields (e.g. high temperatures generally reduce yields, while increased CO_2_ may benefit C3 crops), the real challenge lies in their combined impact. Studies show that co-occurring stress conditions, even at relatively low levels, can severely hinder plant growth and survival when individual conditions alone have milder effects [7–9]. The expected increase in adverse conditions associated with climate change necessitates breeding resilient crops with improved productivity that can be grown on existing arable land.

Plant breeding, which focuses on selecting plants with desirable traits, has long been the foundation of crop improvement. Because desirable traits are typically selected under optimal growing conditions, representative of those under which the crops will be cultivated, this approach may not facilitate the identification of abiotic stress-tolerant genotypes that harbour stress-tolerance genes [10–12]. Plant geneticists have conducted experiments under multiple stress conditions to identify these genes and to distinguish susceptible and tolerant genotypes and analyse DNA-level differences that may be associated with stress resilience [13]. These DNA-level differences, or genetic variants, have been applied in marker-assisted selection to identify genotypes with beneficial alleles linked to abiotic stress, thereby enhancing tolerance in crops like rice and maize [14–16]. Despite the progress in identifying stress-tolerant genotypes and the associated genetic variants, identifying the causal genes remains challenging because abiotic stress tolerance is a quantitative trait and, as such, is controlled by multiple genes and influenced by genotype-by-environment interactions.

Developments in omics technology have enabled researchers to generate multiple types of data (e.g. genomic, transcriptomic, proteomic, metabolomic and phenomic), revealing the molecular processes underlying changes in plant growth and development under diverse conditions. Using these multiomics data, researchers have gained a multidimensional view of how genetic information (i.e. a DNA sequence) is processed to generate functional traits [15–19]. These omics datasets, although containing a wealth of information, are highly heterogeneous. A major challenge lies in integrating data from different sources and formats to generate additional insights. Machine learning (ML) addresses this challenge, as its algorithms can analyse, interpret and create hypotheses from large-scale, heterogeneous data [20].

## 2. Machine learning: a brief overview

**Figure 1.**
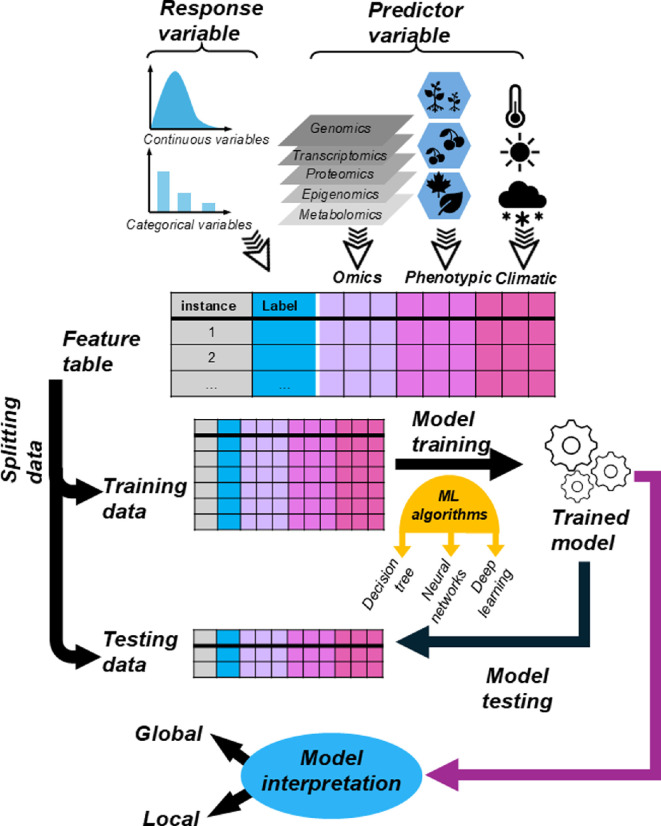
General ML workflow used in supervised approaches. The first step is constructing a feature table, which includes the response variable (label) to be predicted and the predictor variables (features) for each data point (instance). Both the label and feature data can be categorical or continuous values. Multiple feature types (e.g. omics, phenotypic, climatic, imaging, sound) can be used in plant biology-related predictions. The next step is splitting the full dataset into training and testing sets. The training data are used to train models, which are then tested on the testing data to evaluate their ability to predict new, unseen data. If a model performs well on the testing data, it is interpreted using different metrics (such as permutation importances in the case of global interpretation and Shapley additive explanations (SHAP) values in the case of local interpretation) to identify the key features influencing predictions. These features can provide insights into the mechanisms underlying stress responses.

ML involves the development of algorithms that learn from data (*training*) to identify patterns in the form of a *model* [21]. In an ML workflow, the first step is to frame the research question as an ML problem. For example, to identify genes important for drought tolerance, the ML problem is to build a model trained on input data (such as known drought tolerance genes and their expression patterns) to predict whether other genes are important for drought tolerance or not. This is an example of *supervised learning*, where a model is trained to learn patterns from input data (referred to as *features*) linked to a specific outcome of interest (important for drought tolerance or not, referred to as the *label*; figure 1). Other types of ML include unsupervised, semi-supervised and reinforcement learning. Unsupervised learning (e.g. hierarchical clustering, principal component analysis, rule-based data analysis) uses unlabelled data to train the models with the goal of identifying patterns within the datasets [22]. Semi-supervised learning is used when the labelled data are limited and the model is trained using a combination of labelled and unlabelled data [22]. Reinforcement learning mimics human trial-and-error learning; algorithms learn from feedback, interacting with the environment and making the best decision to achieve a task [22]. This review is focused on using supervised learning approaches, and the reader is directed to other in-depth reviews of the use of other ML approaches in plant biology [23–28].

In supervised learning, the first step is to collect features (i.e. predictors), such as k-mers derived from gene sequences, and labels (i.e. responses), such as important for drought tolerance or not, for each known instance (e.g. *Arabidopsis* genes). The dataset is then split into subsets for training and testing. The training data are used to train the model, while the testing data are reserved to evaluate how well the model generalizes to unseen data. Frequently, the training set is further divided into training and validation subsets to fine-tune the model and reduce overfitting (i.e. the model works well for training data but not new cases). In the drought tolerance example, a label is categorical (e.g. important for drought tolerance or not), and this type of ML task is referred to as *classification*. To evaluate a classification model, we assess the consistency between the predicted and true labels using metrics such as the area under the receiver operating characteristic curve (AUC–ROC), the area under the precision-recall curve (AUC–PR) and the F1 score to determine the model performance (see electronic supplementary material, table S1 for descriptions and the pros and cons of various metrics used for evaluating the models). In a *regression* task, the labels are continuous values (e.g. degree of drought tolerance) [22]. Performance of regression models is measured by how well the predicted values correlate with the true values using measures such as the Pearson correlation coefficient and the coefficient of determination.

Once a model reaches acceptable performance, it can be interpreted to obtain insights into what features are important for making the predictions and why some instances are poorly predicted [29,30]. There are two major ways to interpret the model: *global interpretation* and *local interpretation*. Global interpretation strategies, using measures such as permutation importance [31], identify features that contribute to model predictions of most instances, capturing patterns reflective of the input data that influence the model’s decision-making. For example, in a model predicting drought tolerance genes, the important features may be relevant to a high degree of differential expression under drought conditions. In contrast, *local interpretation* strategies reveal the contributions of features and/or feature interactions to the predictions of labels for a specific instance or a small set of instances [29]. Tree-based models such as random forest (RF) can provide local explanations by identifying feature combinations that are important for specific predictions (e.g. how k-mers contribute to the prediction of drought tolerance importance for an individual gene) [32]. Shapley additive explanations (SHAP) is a local interpretation approach that provides a detailed breakdown of feature contributions for individual predictions [33]. Understanding which features or feature interactions are most influential in maximizing model performance is essential for identifying features (e.g. genes) that can be targeted in breeding decisions.

## 3. Identifying genes associated with abiotic stress tolerance

Breeding for abiotic stress tolerance can be facilitated by introducing specific genes from resistant germplasm into elite susceptible cultivars or by modifying endogenous genes through genome editing. However, identifying these genes remains a challenge. Hundreds of genomic regions have been associated with abiotic stress resistance in plants through quantitative trait locus (QTL) mapping and genome-wide association studies (GWAS) [13], underscoring the need to prioritize candidate genes for experimental validation. ML approaches offer advantages in the identification of candidate genes because, by integrating multi-omics data, they enable the detection of complex interactions among genes, pathways and environmental factors [34]. These interactions are crucial because gene functions often rely on them [35], yet traditional methods might overlook this complexity.

One way to assess the utility of various types of existing data in narrowing down the number of candidate genes for further validation is to use causal genes (i.e. those experimentally validated to influence phenotypes) as labels. For example, one study showed that features such as functional categories, polymorphism types and paralogue number variations could correctly predict 80% of the causal genes related to abiotic stresses in *Arabidopsis* and rice [36] (for details on algorithms and parameters used for modelling see electronic supplementary material, table S2), indicating that integrating these data types using ML models can facilitate prioritizing QTL candidate genes for further studies. In another application, RF models for predicting cold-responsive genes in rice, *Arabidopsis* and cotton were established by integrating functional annotations, gene sequences and evolutionary features, achieving AUC–ROC values of 0.67, 0.70 and 0.81, respectively [37]. For context, an AUC–ROC of 0.5 indicates random performance, while 1.0 represents a perfect model. AUC–ROC values between 0.7 and 0.8 are considered acceptable and values above 0.8 are considered excellent [38]. This study also demonstrated the transferability of a cold-responsive gene prediction model trained on data from one cotton species to two other cotton species (AUC–ROC > 0.79). Although model performance can be further optimized, these examples demonstrate that ML can facilitate the discovery of genes involved in plant responses to abiotic stress conditions.

Plants adopt both specific and general response mechanisms to adapt to abiotic stresses [39]. Using ML to identify common genes responding to multiple abiotic stressors and those responding to specific ones can provide insights into the general and specific mechanisms underlying abiotic stress responses in plants. For example, an RF model using gene expression data from 10 *Arabidopsis* accessions exposed to salt, heat, cold and high light stress predicted the stress conditions experienced by the accessions with an accuracy of 0.99 [40], and through model interpretation, three genes that may be important for general abiotic stress responses in *Arabidopsis* and rice were identified [41–43]. In another study, an RF model classified rice plants as experiencing one of 13 abiotic/biotic stress conditions with a 0.99 accuracy using gene expression data [44], demonstrating the ability of RF models to identify gene expression patterns associated with different stresses. Together, these studies illustrate the potential of ML to identify genes involved in both general and specific stress responses, which will facilitate the discovery of stress-tolerance genes for crop improvement.

In addition to identifying stress-tolerance genes, ML has also been applied to uncover genes associated with other physiological processes. For instance, XGBoost regression models based on gene expression profiles predicted nitrogen use efficiency under varying nitrogen conditions with a correlation coefficient (*r*) of 0.79 in maize and 0.65 in *Arabidopsis* [45]. Eight genes important for nitrogen use efficiency prediction were validated through loss-of-function mutants in both species, demonstrating ML’s capacity to uncover novel genetic mechanisms. In another study aimed at identifying genes related to flowering time, an RF model was used on a global collection of 383 *Arabidopsis* accessions, combining genetic variants, gene expression and methylation data as input features [46]. This model yielded a Pearson’s correlation greater than 0.6 between observed and predicted flowering times. Interestingly, the model identified both known and novel genes influencing flowering time, with 9 out of 21 novel genes confirmed to affect flowering in the Col-0 accession through loss-of-function analysis. Local interpretation revealed that the predictive importance of features (e.g. genes) varied across genetic backgrounds, highlighting the genotype-dependent contributions of genes to flowering time. The genotype dependence of gene effects may also explain why most novel genes tested did not affect flowering time: these genes were identified as important for predicting flowering time in a global collection of *Arabidopsis* accessions, but their effects were only tested in a single accession (Col-0). Local interpretation also identified 7186 interactions between features. Among these interactions was an interaction between a transcription factor (TF) and genes that the TF was reported to regulate, suggesting that these interactions may represent potential biological relationships between genes.

In summary, these studies demonstrate that ML models can identify important features for predictions that may be associated with phenotypic variability. The ML models were able to identify known causal genes and gene interactions, as well as novel candidate genes and interactions controlling phenotypic responses. This suggests that although ML models do not perfectly predict causal genes or traits, they can be further interpreted to prioritize genes that are likely associated with stress tolerance, enabling more efficient experimental validation and acceleration of resilient crop development. Future studies should consider integrating multiple datasets to increase the sample size, thereby enhancing the generalizability of the models [44]. Additionally, ML models can identify novel causal genes that have not been previously reported in the literature. The use of loss-of-function mutations in target or related species can help externally validate the genes identified as important for prediction [46].

## 4. Understanding gene regulation

Another way to uncover the genetic mechanisms controlling resilience traits is to elucidate the regulatory network underlying gene responses to abiotic stress. Such regulatory networks consist of *cis*-regulatory elements (CREs) located in regions proximal to genes and trans-regulatory factors like TFs [47].

Gene expression is controlled by multiple genomic factors, including promoters, enhancers and TFs [48]. Integrating various omics data using ML can reveal interactions among these regulatory elements. For instance, an RF model combining putative CREs (pCREs), TF binding sites (TFBSs) and sequence data predicted gene expression in rice under heat and drought stress, with AUC–ROC scores of 0.89 and 0.88, respectively [49]. Surprisingly, coding sequences with high GC and low AT contents were more predictive of gene expression than either pCREs or TFBSs [49]. In another study, cold-responsive gene sequences were used to predict genes responsive to cold in switchgrass, achieving a median F1 score of 0.85 across multiple time points [50]. Models using the top predictive pCREs identified novel cold-responsive elements that outperformed known TFBSs in predicting cold responses, highlighting these pCREs as potential new regulatory regions in switchgrass [50]. This approach was also applied to identify pCREs associated with drought- and heat-responsive expression in *Arabidopsis* [51].

TFBS and chromatin accessibility data have been integrated to model gene expression responses under abiotic stresses. For instance, Song *et al*. [52] used an ensemble model called Condition-Specific Regulatory network inference engine (ConSReg) to combine multiple ML models to predict regulatory genes in *Arabidopsis*, achieving an AUC–ROC of 0.84 when identifying TFs that regulate the expression of genes responsive to cold, heat and drought [52]. Using a similar approach, Gupta *et al*. [53] constructed a gene regulatory network using gene expression data to identify TFs involved in gene regulation under drought stress in rice. Using the network connectivity patterns as features, a support vector machine classifier distinguished drought-responsive TFs, achieving an AUC–PR of 0.81 [53]. Furthermore, they found that the TFs classified as drought-responsive can be divided into two groups: TFs that are specific to rice and TFs that appear to play a conserved role in plant adaptation to drought. This conclusion is supported by the fact that the latter group includes orthologous genes previously reported to function as drought regulators in *Arabidopsis*, barley, maize and sorghum. These studies suggest that combining TF-related data with other genomic and epigenetic layers may reveal the complex regulatory networks underlying abiotic stress response.

In summary, these findings demonstrate that ML models can predict gene expression and identify regulatory elements. However, gene expression regulation is a complex process influenced by factors such as tissue type, cell type, epigenetic modifications and treatments. The studies presented in this section address this complexity in part by utilizing single-cell data [52], different stress conditions [51] and time-series data to identify changes in regulatory elements over time [50,51]. To enhance our understanding of gene regulation, future studies should focus on integrating multiple independent datasets to examine how gene regulation changes across different tissues, time points and treatments. Additionally, it will be important to investigate whether regulatory elements for specific combinations of these factors are conserved across species.

## 5. Designing synthetic promoters

Once the regulation of stress-responsive gene expression is understood, this knowledge can be used to design promoters that drive expression under the desired conditions. Such synthetic promoters consist of specific types and numbers of CREs placed upstream of a native or minimal promoter of a transgene. These promoters have an advantage over native promoters because the strength (i.e. expression level driven by a promoter) and specificity of the promoter can be controlled to achieve the desired level of gene expression [54–56]. Another advantage of synthetic promoters is that they can be designed using randomly selected CREs from various organisms; this minimizes the chance of homologous recombination and epigenetic silencing, which can occur when the same promoter sequences are used for the expression of multiple transgenes [56]. In one noteworthy study, minimal synthetic promoters (MinSyns) with predictable strengths were constructed from CREs identified in plant and pathogen genes [57]. The strengths (i.e. expression level of a downstream reporter gene) of a library of 1000 constitutive MinSyn promoters, which consist of 3−10 CREs placed in a randomly selected order upstream of a core promoter region (TATA box), were predicted by assigning a score to each nucleotide based on the position of the CRE relative to the core promoter. This score was then converted into predicted promoter strength based on the strengths of a subset of MinSyn promoters that were determined experimentally. The predicted strengths of these MinSyns showed a good correlation with the actual expression values (*R*^2^ = 0.71). This suggests that synthetic promoters can be created by placing CREs, regulated by either endogenous or orthogonal TFs, in a specific arrangement in the core promoter region to control the relative expression of output genes [57]. However, constructing promoters using this approach requires knowledge of the CREs and their interacting TFs, and testing all combinations of CREs experimentally is very labour intensive. Synthetic promoters can also be created by introducing mutations in the native promoter region through error-prone PCR, followed by the selection of promoters with increased strength, often referred to as directed evolution [58]. As with the creation of MinSyns, this approach relies on extensive screening of random DNA sequences, which can be labour and resource intensive.

An alternative to testing all CREs experimentally is to use ML to extract important features from the promoter region regulating gene expression; this allows the sequences subjected to experimental screens to be selected in a more efficient and scalable manner [59–61], which can significantly reduce the cost of promoter engineering. Such ML-based approaches have enabled the de novo design of promoters in bacteria [62–64]. For example, an ML-based regression algorithm, called chaos-attention net for promoter evolution (CAPE), was developed to predict the strength of native promoters and the forces driving their evolution. CAPE, which extracts evolutionary information—i.e. sequence similarity between promoters—predicted the strengths of promoters with higher accuracy compared with previous models that lacked such information [62,64]. In experimental validation, around 37.5% of promoters designed using CAPE drove higher expression than the constitutive nisin-induced PnisA promoter in *Lactococcus lactis,* suggesting that such approaches can be used to enhance the expression strength of prokaryotic promoters [62]. A different ML algorithm, a diffusion-based generative model, was developed for the de novo design of promoters in *Escherichia coli* using native promoter sequences and gene expression data [63]. The model generated synthetic promoters that closely resembled native ones and captured essential features, such as the presence of −10 and −35 motifs, GC contents and k-mer frequency, indicating that promoters can be designed by fitting sequence information to gene expression data. ML has also been used to facilitate the design of plant synthetic promoters. Jores *et al*. [65] used reporter assays to measure the activities of promoters from *Arabidopsis* and maize and found that the promoter strength was influenced by the TATA box, promoter GC content and promoter-proximal TF binding sites; synthetic promoters made using this information had activities comparable to the 35S minimal promoter. Next, they used a convulational neural network (CNN)-based modelling approach to predict promoter strength using core promoter sequences as features, yielding *R*^2^ values of 0.71 for tobacco leaf and 0.67 for maize protoplasts. These CNN models were then used for *in silico* evolution of 150 native promoter sequences of *Arabidopsis* and maize, and an increase in promoter strength was obtained after three rounds of evolution [65].

Although the studies published to date have been aimed at increasing promoter strength rather than increasing response to abiotic stress, they highlight the possibility of using ML approaches to uncover novel regulatory features related to abiotic stress response and to design synthetic promoters driving abiotic stress-related gene expression at the desired levels to counter the harmful effects of stress. However, to design such promoters, we still need to understand the syntax of CREs, which is their spacing, order and function within a promoter, and its correlation with gene expression in plants. ML approaches, such as transfer learning, could be used to leverage the knowledge gained from other systems for synthetic promoter design in crop plants.

## 6. Engineering metabolic networks

Plants produce diverse metabolites, including amino acids, sugars, organic acids, flavonoids, terpenoids, alkaloids and phenolic compounds, that are essential for survival under stress conditions [66–69]. Several metabolites, such as gamma-aminobutyric acid, which functions as an alternative energy source [67], antioxidants that protect cells from reactive oxygen species [66] and flavonoids that protect cells from UV-light damage, accumulate in response to abiotic stress [70]. However, there is little information on the genes or pathways involved in the synthesis of such metabolites.

Identifying the genes responsible for synthesizing specific metabolites remains a significant challenge. Plants produce a vast number of specialized metabolites (SMs), and not all species produce the same metabolites. Consequently, knowledge of SM pathways from one species may not be directly transferable to others. Furthermore, the complex interplay between different metabolic pathways makes it difficult to isolate individual pathways for metabolic engineering [71,72]. Traditional approaches used to identify metabolic genes and pathways, such as gene annotation, evolutionary conservation analysis, co-expression network analysis, protein domain analysis and metabolic GWAS (reviewed in [71,73]) have been helpful but often limited in their predictive power, as they rely on a single dataset. ML offers a promising alternative: integration of multi-omics data to improve predictions and identify SM genes and pathways across various crop species [74,75].

One example of the use of ML models in SM gene prediction is the prediction of the functional annotations of unclassified plant genes. Using multi-omics data from *Arabidopsis*, tomato and maize, Bai *et al*. [74] built an ensemble model (i.e. a model that aggregates predictions from multiple models to improve accuracy and robustness) that predicted SM genes involved in the biosynthesis of terpenoids, alkaloids and phenolics within and across species using proteomics and genomics features. Their model correctly classified 92% of genes within the species from which the training data originated and achieved up to 78% accuracy when models trained on data from one species were applied to another species. In another study focused on *Arabidopsis*, multi-omics data enabled highly accurate prediction of SM genes and genes for general metabolites (i.e. metabolites important for fundamental processes; AUC–ROC = 0.87) [76], with about 50% of discrepancies between predictions and annotations potentially due to gene misannotations. ML shows potential in two areas of SM gene prediction: identifying uncharacterized SM genes across species, particularly in data-poor species, and identifying potential misannotations that prevent elucidating the molecular basis of SM.

Along with identifying SM genes, reconstructing metabolite pathways is also important. Metabolic pathway reconstruction is based on experimental evidence and computational predictions using data, such as annotated genomes and information from known pathways found in reference databases such as PlantCyc [77], KEGG [78] and BioCyc [79–81]. In one study, ML was combined with metabolite network correlation analysis to identify novel pathways in tomatoes [75]. By mapping metabolites of known tomato and non-tomato pathways as subgraphs onto metabolite correlation networks and computing network features for each subgraph, an ML model was generated to classify pathways as either a tomato or non-tomato pathway. This model showed high performance (AUC–ROC = 0.93) in predicting the presence of pathways such as ß-alanine-degradation and tryptophan-degradation through indole pyruvate pathways, which were previously not known to be present in plants, suggesting that ML can help in the identification of unknown pathways in plants [75]. In another study, Bao *et al*. [81] used the Graph Transformer and CNN model to predict plant SM pathways and classify natural products such as alkaloids and phenylpropanoids. Their model outperformed other models trained on KEGG datasets and showed an average prediction accuracy of 98% [81].

The major problem with metabolic pathway reconstruction is that the reference databases currently available for metabolic pathways are based on known gene annotations and ontology and thus can identify only known metabolic pathways. Therefore, future strategies should involve not only the identification of SM genes but also the discernment of how those genes produce a specific metabolite. This is important since the knowledge obtained from one species may not be transferable to other species [68,69], and current ML models are trained on reference databases, which may not have information from all crop species.

## 7. Making photosynthesis more efficient

Photosynthesis is the primary pathway that fixes CO_2_ from the atmosphere and is the basis of crop production. Global warming, characterized by increased CO_2_ and higher temperatures, consistently reduces photosynthesis and, thus, the yield of crop plants [82]. To address this challenge, researchers are exploring synthetic biology strategies facilitated by ML to enhance photosynthetic efficiency [83–85].

One strategy is to make the CO_2_-fixation step more efficient: up to 30% of fixed CO_2_ in plants is wasted through photorespiration (when Rubisco reacts with O_2_ instead of CO_2_), and the rate of this energetically wasteful reaction increases with increasing temperature [86]. Attempts to engineer Rubisco with lower affinity to O_2_ have had limited success [87]. Thus, researchers have raised the possibility of exploiting the CO_2_-fixing ability of other enzymes present in at least eight different microorganisms that may perform the carboxylation reaction [85,88,89]. Synthetic biology, coupled with ML, provides an exciting opportunity to design new CO_2_-fixing pathways using enzymes with improved kinetic and thermodynamic efficiencies [85]. For instance, ML-guided protein engineering was used to improve the efficiency of a new-to-nature enzyme, glycolyl-CoA carboxylase (GCC), which catalyses the carboxylation reaction in a synthetic photorespiration bypass (the tartonyl-CoA pathway), boosting the CO_2_ uptake rates by 20–60% in *in vitro* reactions [90]. In addition, using data from enzyme activity assays on randomly mutagenized versions of GCC, ML models were trained to predict promising mutations for further testing, demonstrating how ML can streamline the screening of enzyme variants for metabolic engineering applications [90].

Another example involves improving the crotonyl-coenzyme A (CoA)/ethylmalonyl-CoA/hydroxybutyryl-CoA (CETCH) cycle, which incorporates novel CO_2_-fixing enzymes called enoyl-CoA carboxylases/reductases from α-proteobacteria and *Streptomyces*, to fully replace endogenous photosynthesis in *in vitro* reactions [91]. These enzymes are oxygen insensitive and more catalytically efficient than Rubisco. The CETCH cycle consists of 17 different enzymes obtained from nine different organisms and 10 cofactors, and the resulting pathway is five-fold more efficient than most of the endogenous carbon fixation pathways. Since these enzymes are from different organisms, their activities need to be optimized. This would involve testing the synthetic CETCH cycle under 10^25^ conditions in wet lab experiments. To overcome this challenge, an ML screening strategy was developed to use iterative design–build–learn cycles to explore various reaction combinations. This significantly reduced the number of experiments required, and a 10-fold increase in CO_2_-fixation efficiency compared with the original cycle was obtained after just five rounds of optimization [92]. Although the CETCH cycle has yet to be incorporated into plants, it has been assembled to work in chloroplast extracts, forming an artificial chloroplast [93].

Although these studies demonstrate how ML can significantly reduce the time and effort needed to screen thousands of enzymes and experimental conditions for increased photosynthesis efficiency, the strategies have yet to be tested in plants. The above-mentioned examples show that strategies exist to overcome the limitations of natural photosynthesis. By combining ML-based optimization and synthetic biology, it is possible to develop crop plants with enhanced photosynthetic efficiency under adverse conditions.

## 8. Predicting plant responses under stress

Plant responses to stress are manifested as changes in phenotypes, such as leaf colouration and emission of volatile compounds or even sounds [94–96]. High-throughput plant image data documenting changes in response to diverse stress conditions can be analysed using ML to quantify the severity of stress before any visible symptoms appear in field settings [97–99]. For example, ML algorithms have been developed to classify multiple crop species as stressed or unstressed under drought conditions [100–104]. Kaneda *et al.* developed a model integrating environmental data (e.g. transpiration rate) and plant images to predict water stress levels in plants [101]. This model utilized a deep neural network with an image feature extractor to identify wilting based on plant motion. The multi-modal model significantly outperformed models that rely solely on image data [101]. In another study, Das Choudhury and colleagues [102] used a deep neural network called HyperStress Propagate Net using hyperspectral imagery data as features to predict the onset of drought and classify cotton plants as stressed or unstressed (F1 score = 0.98). This algorithm was able to detect the early effects of drought stress, within 3 days of onset, by comparing the reflectance spectra at different wavelengths generated from hyperspectral imagery of stressed and control plants, indicating that stress can be detected before visible symptoms appear [102]. In a recent study, ultrasonic airborne sounds emitted from tomato and tobacco plants were found to reflect the physiological status of the plants [96]. ML models trained on these acoustic emissions could distinguish between drought-stressed, injured and control plants. For example, ML models could distinguish between high and low levels of dehydration stress in tomato plants under greenhouse conditions with an accuracy of 81%, suggesting that sounds emitted from plants can be utilized to monitor their drought stress levels [96].

These examples demonstrate that ML models can effectively identify patterns in high-throughput plant phenotyping data and reveal signs of stress, even before any visible symptoms appear. This application is important because it enables timely intervention to maintain plant health under adverse conditions, which is a crucial part of improving crop resilience. However, to design resilient crops the focus should be on studying multiple abiotic stresses. Recently, studies have focused on incorporating multi-omics data from multiple abiotic stresses, such as a recent study in potato [105] where high-throughput phenotyping and multi-omics data analysis were carried out under heat, drought and water-logging conditions. These data will be useful to include in future ML training models.

## 9. Approaches for designing resilient crops and challenges

The integration of extensive multi-omics data with interpretable ML models holds immense potential to unravel the biological mechanisms underlying resilient crop traits. Examples in the previous sections demonstrated the successful application of ML models in predicting molecular functions and complex phenotypic–environmental interactions, advancing our understanding of the genetic and mechanistic bases of crop resilience. However, a critical challenge remains: translating this knowledge into the development of climate-resilient crops.

**Figure 2.**
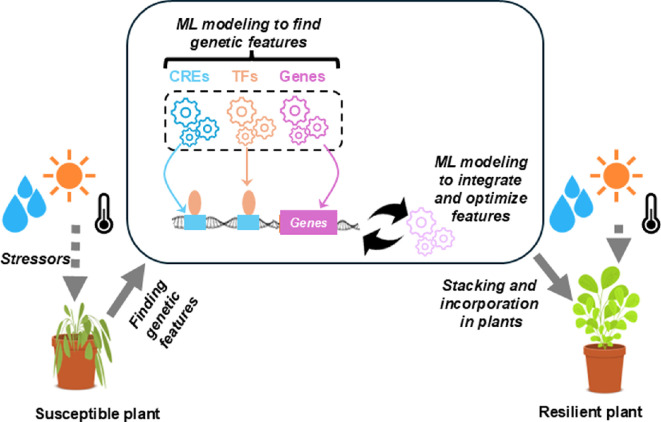
Designing a resilient plant. A plant susceptible to climatic conditions can be engineered to have genetic features that increase resilience. Such features include CREs, TFs and genes related to abiotic stress tolerance that are identified with the help of ML approaches. ML modelling can help determine the optimal combination of traits that can be stacked together in plants. These traits can then be incorporated into plants either through transformation or the use of genome editing tools, leading to the development of a resilient plant.

Introducing a single gene conferring tolerance to abiotic stresses has yielded promising results in some cases (reviewed in [106,107]), such as increased drought resistance through the expression of a vacuolar H^+^pyrophosphatase in maize [108] and increased thermotolerance obtained by expressing *Thermo-tolerance 1* in rice [109]. However, plants are exposed to multiple abiotic stresses simultaneously in the field, and the responses to these stresses might involve different pathways that either enhance or compromise stress resistance. In addition, we should also consider the effect of increasing stress resilience on yield. Trade-offs between resilience and yield arise because plants have finite resources that must be allocated among various physiological processes, including growth, reproduction and defence [110]. To maintain yield under stress, plants must balance resource use between processes that enhance resilience and those that support yield. Consequently, when designing stress-resistant crops, accounting for this plasticity of resource allocation is crucial to ensure stable yields under varying environmental conditions [110]. Examples in the previous sections provide evidence that ML models can identify genes and regulatory elements associated with resilience; this information can be combined with genes associated with yield in multiple species [111–114] to support the development of resilient crop cultivars with minimal yield penalties. One way to balance resilience and yield is by ensuring that stress-responsive traits are expressed only when needed, thereby preserving resources for yield under normal conditions and mitigating potential penalties associated with constant trait expression. Creating crops that express stress-responsive traits precisely when needed requires a comprehensive understanding of gene expression, metabolic pathways and regulatory modules. This integrated approach, as illustrated in figure 2, involves the identification of crucial genes, such as those conferring stress resistance, and introgressing them into susceptible germplasm. Such genes can be regulated by a synthetic genetic circuit composed of CREs and corresponding TFs that activates or represses expression in response to environmental cues. Multiple genes, each controlled by a genetic circuit, can be stacked to create a robust stress response against multiple abiotic and biotic stresses. Genome editing tools, such as CRISPR/Cas9, can be used to create and transfer these modules into plants. ML models can greatly accelerate these tasks by helping to identify optimal combinations of features, such as genes and regulatory elements, that need to be incorporated in a plant for specific stress conditions.

Although ML models have recently been useful in elucidating various aspects of stress-tolerance mechanisms, several shortcomings need to be addressed for making efficient use of ML. One major limitation is the scarcity of experimentally validated causal genes for model validation. In such cases, semi-supervised learning can be used, for example, to mine functional genes in data-scarce species [115]. Some studies reviewed here have overcome this problem by using loss-of-function mutants or information from related species to validate the effects of genes on target traits. Another significant challenge is the limited sample size in most studies, which reduces an ML model’s ability to discover generalizable patterns in data that can reveal the genetic mechanisms of resilience traits. An effective approach to increase sample size involves integrating multiple independent datasets, which can enhance the generalizability of the models. A second critical challenge is the lack of genomic annotations and omics data for many crops, which reduces the performance of ML models. Transfer learning, in which models pre-trained on large, well-annotated datasets, such as those from *Arabidopsis*, and fine-tuned for crops with smaller datasets, such as rice or maize, is a potential solution for elucidating abiotic stress-tolerance mechanisms in data-scarce species [116]. However, even in the model plant *Arabidopsis*, only 74% of proteins are associated with a gene ontology term, and only half of these proteins (31−38%) have terms supported by experimental evidence [117]. This knowledge gap must be addressed, and ML can assist by identifying functional annotations from the literature [117–119]. A third challenge is understanding cell-specific responses to environmental stresses, which can be masked when analysing data from multiple tissue types. In this regard, single-cell data can provide high-resolution insights into how individual cells respond to abiotic stresses. However, these datasets are often sparse and complex owing to high dimensionality (i.e. a large number of features, such as gene expression data, relative to the number of samples) and variability across cell types [120]. Such challenges can be addressed by using advanced ML models, such as deep-learning techniques, to identify underlying cell patterns and relationships [121]. Furthermore, integrating single-cell data with multi-omics data, such as epigenomics data, could enhance our understanding of how abiotic stress tolerance is regulated at the cellular level. Integration of such datasets and clustering of single-cell omics data can be performed using unsupervised learning [122]. A fourth challenge lies in understanding interactions across different omics datasets. One approach is to use graph-based ML models, which offer a framework for identifying genetic mechanisms associated with abiotic stresses by representing biological data as networks in which, for example, entities such as genes or proteins are represented as nodes and their interactions or regulatory relationships are depicted as edges [123]. By capturing relationships within and across data layers, graph-based approaches are well-suited for studying the interconnected nature of plant stress responses [124], the elucidation of which will further advance our understanding of abiotic stress tolerance.

Together, ML approaches, including transfer learning, single-cell modelling and graph-based methods, address key challenges in understanding abiotic stress tolerance. They provide powerful tools to uncover the genetic, molecular and cellular mechanisms underlying stress resilience, offering a clear pathway towards more effective crop improvement strategies.

## 10. Conclusion

Designing stress-resilient crops will require a comprehensive understanding of the various processes affecting plant growth, such as gene expression, metabolic pathways and regulatory modules. ML, a data-driven approach that uses algorithms to analyse large datasets to identify patterns and trends, offers a promising avenue to enhance our understanding of mechanisms controlling abiotic stress tolerance in plants. By integrating high-dimensional data, ML aids in identifying candidate genes, regulatory elements and pathways associated with resilience traits. Moreover, its ability to analyse spectral and image data allows for precisely detecting stress indicators, enhancing the efficiency and accuracy of large-scale phenotyping in experimental settings. Importantly, insights from ML models trained on species with extensive data can be transferred to related species with limited data. This underscores ML’s potential to contribute significantly to the breeding and development of stress-tolerant varieties of crops for which there are less data available.

## Data Availability

Supplementary material is available online [125].
